# Cardiorespiratory fitness in COPD and HF from the Fitness Registry and the Importance of Exercise: a National Database

**DOI:** 10.1093/ehjopen/oeae104

**Published:** 2024-12-17

**Authors:** Jacinthe Boulet, Jonathan Myers, Jeffrey W Christle, Ross Arena, Leonard Kaminsky, Anna Nozza, Joshua Abella, Michel White

**Affiliations:** Division of Cardiology, Department of Medicine, Université de Montréal, Montreal Heart Institute, 5000 Belanger Street, Montreal, Quebec, Canada H1T 1C8; Cardiology Division, Veterans Affairs Palo Alto Health Care System/Stanford University, Palo Alto, CA 94304, USA; Division of Cardiovascular Medicine, Department of Medicine, Stanford University, Stanford, CA 94305, USA; Department of Physical Therapy, College of Applied Health Sciences, University of Illinois at Chicago, Chicago, IL 60612, USA; Fisher Institute of Health and Well-Being, Ball State University, Muncie, IN 47306, USA; Montreal Health Innovations Coordinating Center, Montréal, Quebec, Canada H1T 1C8; Cardiology Division, Veterans Affairs Palo Alto Health Care System/Stanford University, Palo Alto, CA 94304, USA; Division of Cardiology, Department of Medicine, Université de Montréal, Montreal Heart Institute, 5000 Belanger Street, Montreal, Quebec, Canada H1T 1C8

**Keywords:** Exercise physiology, Respirology, Congestive heart failure, Cardiomyopathy, Risk factors

## Abstract

**Aims:**

To better characterize functional consequences of the presence of COPD on cardiorespiratory fitness in patients with HF.

**Methods and results:**

Patients with any clinical indication for cardiopulmonary exercise testing (CPET) were included in the international FRIEND registry. Diagnosis of COPD was confirmed by a ratio of forced expiratory volume in 1 s and forced vital capacity (FEV_1_/FVC) < 0.70. HF was diagnosed in the presence of symptoms and signs of HF. A total of 10 957 patients were divided into four groups: patients without HF or COPD (*n* = 8963), patients with HF (*n* = 852) or COPD (*n =* 991) alone, and patients with both HF and COPD (*n* = 151). Maximal workload was the lowest in patients with both HF and COPD [78.09 (95% CI: 72.92, 83.64 watts)], and all pairwise comparisons with adjusted *P* < 0.05 between groups were statistically significant. Patients with both HF and COPD yielded the lowest PETCO_2_ values [31.80 (95% CI: 31.00, 32.60)] mmHg and exhibited a higher VE/VCO_2_ slope compared with HF (36.73 (95% CI: 35.78, 37.68) vs. 33.91 (95% CI: 33.50, 34.33 units, *P*  *<* 0.0001). Peak VO_2_ was the lowest with concomitant HF and COPD 19.93 (95% CI: 18.60, 21.27) mL/kg/min and was significantly different compared with all other groups (*P* < 0.05).

**Conclusion:**

Patients referred for CPET with COPD and concomitant HF exhibit a profound impairment in CRF compared with patients with COPD or HF alone. Early identification of pulmonary obstruction in patients with HF by more frequent usage of pulmonary function testing may contribute to providing better treatment for both COPD and HF in these high-risk individuals.

## Introduction

Heart failure (HF) is a growing healthcare concern in the civilized countries with at least 26 million people worldwide affected by the disease.^1^ HF is characterized by impairment in both maximal and submaximal exercise capacity.^2^ Moreover, the magnitude of impairment in both peak VO_2_ and submaximal exercise time (ET) assessed by various testing modalities has been associated with adverse outcomes in these high-risk patients.^2^

The presence of chronic obstructive pulmonary disease (COPD) is estimated to be between 20% and 40% in the HF population.^3^ Pulmonary obstruction has significant haemodynamic consequences, such as increased pulmonary vascular resistance, pulmonary hypertension, and reduced right ventricular function.^4,5^ Similarly, COPD and the presence of lung hyperinflation are associated with a reduction in cardiac end-diastolic and end-systolic stroke volumes.^5,6^ When associated with HF, the impairment in pulmonary and cardiac reserve may lead to increased adverse events in these patients.^7^ Indeed, this combination of COPD and HF has had a significant impact on prognosis, with COPD being an independent predictor of all-cause mortality and cardiovascular hospitalizations in patients with HF compared with patients with HF without concomitant COPD.^7^ Exercise responses in COPD or HF have been well investigated, but less is known regarding the clinical features and the potential functional consequences of the coexistence of both diseases in a large cohort of unselected patients referred for CPET.

In 2014, a national fitness registry was initiated by the American Heart Association, which involved an ongoing multicentre database that established normative cardiorespiratory fitness (CRF) values in men and women [termed the Fitness Registry and the Importance of Exercise: A National Database (FRIEND)].^8,9^ Using data from the FRIEND registry, the largest database on this matter, the current study reports on the clinical characteristics, CRF, and response to exercise in a large cohort of patients who have COPD and HF.

## Methods

This study was a retrospective analysis from the international FRIEND registry. Enrolment was approved by the Stanford University Institutional Review Board and all the participating centres around the world, and a written consent was obtained from the study participants. The inclusion criteria were patients older than 18 years of age who had performed CPET between 1 January 2008 and 31 December 2013. The indication for CPET was decided upon individual physician clinical judgment. A previous diagnosis of HF was adjudicated as per ongoing current guidelines at the time of the recruitment,^10^ and the diagnosis of COPD was established by a calculated FEV1/FVC ratio < 0.70. Patients with HF were included in the FRIEND database regardless of their LV ejection fraction. CPET was performed on a treadmill with an integrated metabolic cart using breath-by-breath data capture and analysis.^11^ During the last phase of exercise, peak VO_2_ was obtained as the highest average VO_2_ over a 20–30-s period.^12^ All ventilatory metrics (e.g. VE, VCO_2_) were measured continuously throughout the entire exercise period and analysed on a breath-by-breath basis. These metrics were used to obtain the VE/VCO_2_ slope via least squares linear regression (y = mx + b, m = slope).^13^ The achievement of a respiratory exchange ratio (RER; VCO_2_/VO_2_) ≥ 1.05 and rating of perceived exertion (RPE; Borg scale 6–20) of ≥16 were required to designate peak effort. PETCO_2_ was calculated as previously reported.^14^ In accordance with the principles outlined in the Declaration of Helsinki, our research involving human subjects was conducted with a commitment to ensuring the highest standards of ethical integrity, prioritizing the health, rights, and dignity of the participants throughout the study.

### Statistical methods

Continuous variables are presented as mean ± standard deviation (SD), and categorical variables are presented as counts (proportions). An analysis of covariance (ANCOVA) was performed, respectively, on the following outcomes: maximal workload, VO_2_ peak, PETCO_2_, max VE, and VE/VCO_2_ slope with terms for group (Group 1: < 0.7 COPD, HF = 0; Group 2: < 0.7 COPD, HF = 1; Group 3: ≥0.7 no COPD, HF = 0; Group 4: ≥0.7 No COPD, HF = 1), gender, age and beta-blockers (BB) use as covariates. The covariates were chosen based on their association with the outcomes. Under the ANCOVA model, the adjusted mean for the outcomes was compared between groups. The required assumptions underlying the planned model (normality of residuals, equality of variances between groups) were checked for validity. The outcome MaxLoad did not respect the normality of the residuals, therefore a logarithm transformation of the data was done, and geometric means are reported. No adjustment was made for multiple comparisons. All statistical analyses were performed using SAS 9.4.

## Results

The clinical characteristics of the study population are presented in *Table 1*. A cohort of 10 957 patients had complete data and were divided into four groups as detailed above. Patients with HF were significantly older. There was a higher rate of use of statins, antihypertensive medications, angiotensin-converting enzyme inhibitors or angiotensin receptor blockers, BB, digoxin, diuretics, and nitrates in patients with HF compared with patients without HF.

**Table 1:** Clinical characteristics of the study population

Characteristics	No COPD–no HF	COPD–no HF	HF–no COPD	COPD–HF
Total (*n* = 10 957)				
*n*	8963	991	852	151
Age (y)	47 ± 13	53 ± 14	54 ± 14	65 ± 12
Females (*n*%)	2074 (23)	189 (19)	307 (36)	25 (17)
Males (*n*%)	6889 (77)	802 (81)	545 (64)	126 (83)
BMI (kg/m²)	28 ± 5	27 ± 4	30 ± 6	28 ± 5
Waist (cm)	90 ± 15	91 ± 15	95 ± 15	99 ± 11
Body fat (%)	30 ± 10	28 ± 10	32 ± 10	30 ± 8
Smoking history (*n*%)	752 (8)	161 (16)	261 (31)	73 (48)
CABG (*n*%)	66 (1)	14 (1)	77 (1)	34 (23)
PCI (*n*%)	100 (1)	21 (2)	101 (12)	30 (20)
MI (*n*%)	62 (1)	13 (1)	152 (18)	52 (34)
Hypertension (*n*%)	2605 (29)	299 (3)	400 (47)	90 (60)
Diabetes (*n*%)	263 (3)	35 (4)	168 (20)	32 (21)
Any CVD (*n*%)	367 (4)	83 (8)	613 (72)	139 (92)
Cancer (*n*%)	124 (1)	21 (2)	47 (6)	8 (5)
Statin (*n*%)	1519 (17)	183 (18)	247 (29)	77 (51)
Antihypertensives (*n*%)	521 (6)	113 (11)	209 (25)	69 46)
ACEI/ARB (*n*%)	345 (4)	63 (6)	328 (38)	93 (62)
BB (*n*%)	816 (9)	126 (13)	453 (53)	107 (71)
CCB (*n*%)	229 (3)	43 (4)	65 (8)	18 (12)
Digoxin (*n*%)	13 (0.1)	1 (0.1)	132 (15)	32 (21)
Diuretics (*n*%)	435 (5)	69 (7)	399 (47)	101 (67)
Nitrate (*n*%)	217 (2)	19 (2)	101 (12)	19 (13)

ARB, angiotensin receptor blocker; ACEI, angiotensinogen-converting enzyme inhibitor; BB, beta-blocker; BMI, body mass index; CABG, coronary artery bypass graft; CCB, calcium channel blocker; cm, centimetre; CVD, cerebrovascular disease; kg, kilogram; m, metre; MI, myocardial infarction; PCI, percutaneous coronary intervention. Continuous characteristics are expressed as means ± SD and categorical characteristics as counts (percentages).

Selected haemodynamics, exercise, and pulmonary function characteristics are given in *Table 2*. Resting heart rate, systolic blood pressure (SBP), and diastolic blood pressure were similar for all four groups. Group 4 exhibited a shorter ET, lower peak heart rate, and SBP. Additionally, both FEV1 and FVC mean values were significantly lower in the COPD—HF group compared with COPD alone.

**Table 2:** Resting haemodynamics, exercise parameters, and pulmonary function data for the study population

Characteristics	No COPD–no HF (Group 1)	COPD–no HF (Group 2)	HF–no COPD (Group 3)	COPD–HF (Group 4)
Baseline parameters				
HR (b.p.m.)	72.30 ± 0.23	72.36 ± 0.46	71.90 ± 0.48	73.61 ± 1.09
SBP (mmHg)	124.77 ± 0.30	125.67 ± 0.59	120.33 ± 0.62	118.08 ± 1.40
DBP (mmHg)	78.16 ± 0.18	77.20 ± 0.36	75.25 ± 0.39	74.17 ± 0.94
Pulmonary parameters				
FEV_1_ (L)	3.06 ± 0.01	2.50 ± 0.02	2.85 ± 0.02	2.17 ± 0.05
FVC (L)	3.78 ± 0.01	3.93 ± 0.03	3.53 ± 0.03	3.36 ± 0.06
FEV_1_/FVC (%)	0.809 ± 0.001	0.638 ± 0.002	0.809 ± 0.002	0.640 ± 0.005
Exercise parameters				
ET (min)	9.47 ± 0.06	8.94 ± 0.13	9.67 ± 0.10	8.89 ± 0.23
VT (mL/min/kg)	15.15 ± 0.10	15.04 ± 0.20	16.67 ± 0.21	16.05 ± 0.58
Peak HR (b.p.m.)	151.31 ± 0.34	149.51 ± 0.69	146.56 ± 0.72	139.70 ± 1.63
HR at VT (b.p.m.)	114.01 ± 0.35	112.53 ± 0.71	117.57 ± 0.66	116.64 ± 1.75
Peak SBP (mmHg)	174.81 ± 0.45	172.67 ± 0.94	159.14 ± 0.95	149.19 ± 2.15
Peak DBP (mmHg)	82.27 ± 0.22	82.22 ± 0.45	78.36 ± 0.47	77.59 ± 1.08
Peak RER (units)	1.158 ± 0.002	1.136 ± 0.003	1.135 ± 0.004	1.107 ± 0.008
Peak RPE (units)	18.03 ± 0.05	17.89 ± 0.12	17.54 ± 0.08	16.97 ± 0.20

DBP, diastolic blood pressure; ET, exercise time; FEV1, forced expiratory volume in the first second; FVC, forced vital capacity; HR, heart rate; MI, myocardial infection; PCI, percutaneous coronary intervention; RER, respiratory exchange ratio; RPE, rate of perceived exertion; SBP, systolic blood pressure; VT, ventilatory threshold. Continuous parameters are expressed as means ± SD and categorical parameters as counts (percentages).

**Figure 1:**
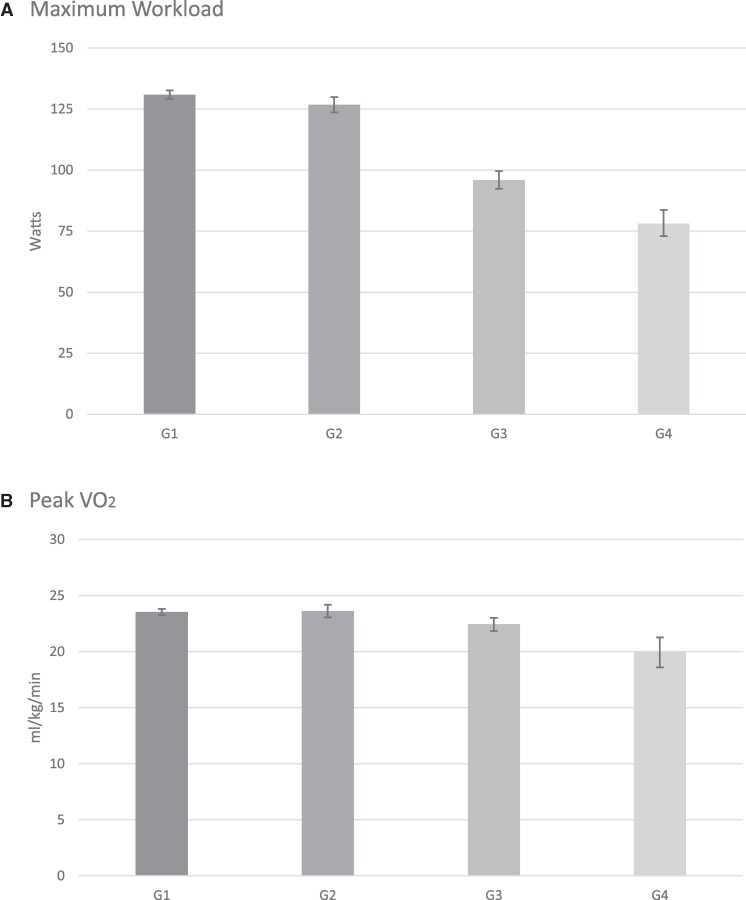
Results for maximal workload (*A*) are presented as adjusted geometric means (including their 95% CI). All other results are presented as adjusted means (95% CI). Values are given in *Table 3*. For maximal workload, all pairwise comparisons were significant with *P* < 0.0001 except for Group 1 vs. 2 with *P* = 0.0067. For peak VO_2_ (*B*), all pairwise comparisons were significant at *P* < 0.005 except for Group 1 vs. Group 2 with *P* = 0.7337. VO_2_, oxygen uptake.

**Figure 2:**
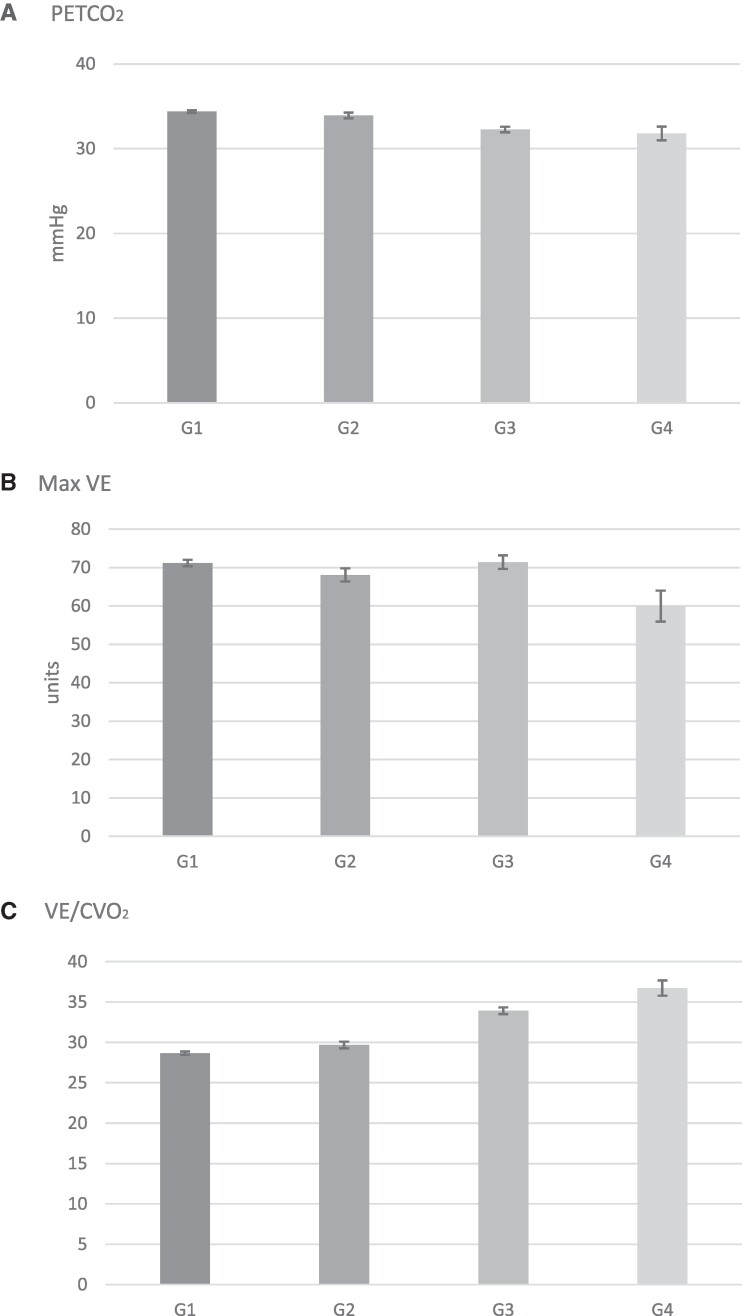
Results are presented as adjusted means (95% CI). Values are given in *Table 3*. For PETCO_2_ (*A*), all pairwise comparisons were significant at *P* < 0.0001 except for Group 1 vs. 2 with *P* = 0.0058 and Group 3 vs. 4 with *P* = 0.2936. For maximal VE (*B*), all pairwise comparisons were significant with *P* < 0.05 except for Group 1 vs. 3 with *P* = 0.8386 and Group 2 vs. 3 with *P* = 0.0075. For VE/VCO_2_ slope (*C*), all pairwise comparisons were significant at *P* < 0.0001. PETCO_2_, partial pressure of end-tidal carbon dioxide; VE, minute ventilation; VE/VCO_2_, minute ventilation over carbon dioxide production slope.

Between-group comparisons for the four groups for selected ventilatory and exercise parameters are shown in *Figures 1* and *2*. Maximal workload and peak VO_2_ are presented in *Figure 1*. In patients with non-HF, the presence of COPD was associated with a small yet significantly lower maximal workload compared with patients without COPD 78.09 (95% CI: 72.92, 83.64) watts (Group 4) vs. 95.90 (95% CI: 92.33, 99.60) watts (Group 3), (*P* < 0.0001). In the absence of COPD, the magnitude of watts achieved was significantly lower in patients with HF compared with patients without HF (*P* < 0.0001). Patients with COPD and HF exhibited the lowest workload, achieving 95.90 (95% CI: 92.33, 99.60) watts (Group 3) vs. 78.09 (95% CI: 72.92, 83.64) watts (Group 4) (*P* < 0.0001). As expected, the differences in peak VO_2_ for the four groups of patients were of similar magnitude as the maximal workload achieved, with the highest VO_2_ achieved among subjects in Groups 1 and 2 23.53 (95% CI: 23.25, 23.81) and 23.62 (95% CI: 23.06, 24.19) (mL/kg/min), respectively, and the lowest VO_2_ observed in patients in Group 4 19.93 (95% CI: 18.60, 21.27) (mL/kg/min). Patients with HF exhibited a significantly lower peak VO_2_ whether they had a concomitant diagnosis of COPD or not 22.44 (95% CI: 21.85, 23.02) mL/kg/min (Group 3) vs. 19.93 (95% CI: 18.60, 21.27) (Group 4) (*P* = 0.0007).

**Table 3:** CPET outcomes adjusted for age, sex, and BB use

Characteristics	No COPD–no HF (Group 1)	COPD–no HF (Group 2)	HF–no COPD (Group 3)	COPD–HF (Group 4)	Pairwise comparison with adjusted *P*-value at *P* < 0.05
Maximal workload (watts)	130.88 (129.13, 132.65)	126.72 (123.64, 129.88)	95.90 (92.33, 99.60)	78.09 (72.92, 83.64)	1 vs. 3: *P* < 0.0001 2 vs. 4: *P* < 0.0001 2 vs. 1: *P* = 0.0067 4 vs. 3: *P* < 0.0001 1 vs. 4: *P* < 0.0001 2 vs. 3: *P* < 0.0001
Peak VO_2_ (mL/kg/min)	23.53 (23.25, 23.81)	23.62 (23.06, 24.19)	22.44 (21.85, 23.02)	19.93 (18.60, 21.27)	1 vs. 3: *P* = 0.0011 2 vs. 4: *P* < 0.0001 2 vs. 1: *P* = 0.7337 4 vs. 3: *P* = 0.0007 1 vs. 4: *P* < 0.0001 2 vs. 3: *P* = 0.0042
PETCO_2_ (mmHg)	34.39 (34.22, 34.55)	33.93 (33.59, 34.27)	32.26 (31.93, 32.59)	31.80 (31.00, 32.60)	1 vs. 3: *P* < 0.0001 2 vs. 4: *P* < 0.0001 2 vs. 1: *P* = 0.0058 4 vs. 3: *P* = 0.2936 1 vs. 4: *P* < 0.0001 2 vs. 3: *P* < 0.0001
Max VE (units)	71.20 (70.36, 72.03)	68.07 (66.36, 69.78)	71.40 (69.65, 73.16)	59.95 (55.90, 63.99)	1 vs. 3: *P* = 0.8386 2 vs. 4: *P* = 0.0003 2 vs. 1: *P* = 0.0002 4 vs. 3: *P* < 0.0001 1 vs. 4: *P* < 0.0001 2 vs. 3: *P* = 0.0075
VE/VCO_2_ slope	28.66 (28.46, 28.87)	29.67 (29.25, 30.09)	33.91 (33.50, 34.33)	36.73 (35.78, 37.68)	1 vs. 3: *P* < 0.0001 2 vs. 4: *P* < 0.0001 2 vs. 1: *P* < 0.0001 4 vs. 3: *P* < 0.0001 1 vs. 4: *P* < 0.0001 2 vs. 3: *P* < 0.0001

Results for maximal workload are presented as adjusted geometric means (including their 95% CI). All other results are presented as adjusted means (95% CI). The *P*-value for each pairwise comparison is also presented.

The data computed for PETCO_2_, max VE, and VE/VCO_2_ slope for the study population are presented in *Figure 2*. In patients with non-HF, the presence of COPD was associated with a close to statistically different lower resting PETCO_2_ compared with patients without COPD 34.39 (95% CI: 34.22, 34.55) mmHg (Group 1) vs. 33.93 (95% CI: 33.59, 34.27) mmHg (Group 2) (*P* = 0.0058). Compared with patients with non-HF, the HF group exhibited a lower PETCO_2_ in both the absence and presence of COPD (*P* < 0.0001). The lowest PETCO_2_ values were observed in patients with COPD and HF compared with patients with HF alone 32.26 (95% CI: 31.93, 32.59) mmHg (Group 3) vs. 31.80 (95% CI: 31.00, 32.60) mmHg (Group 4) (*P* = 0.2936) although this difference was not statistically significant. Maximal VE was statistically significantly lower in patients with both HF and COPD (Group 4) compared with all three groups at 59.95 units (95% CI: 55.90, 63.99) (*P* < 0.0001 vs. Groups 1 and 3, *P* = 0.0003, vs. Group 2). Lastly, the VE/VCO_2_ slope was most elevated in patients with both HF and COPD 36.73 (95% CI: 35.78, 37.68) units exhibiting a statistically significant difference with all other groups (*P* < 0.0001).

## Discussion

The present study describes the clinical and CPET characteristics of patients with and without COPD or HF in a large cohort of healthy subjects and in patients with CPET performed for various clinical indications. We observed that patients with both HF and COPD were slightly older and demonstrated a marked decrease in maximal exercise capacity with several parameters consistent with a significantly greater respiratory inefficiency. These real-life observations from the large FRIEND database represent, to our knowledge, the largest study assessing at demographics and CPET parameters in an unselected population of patients with COPD with and without HF.

CPET has been shown to be a reliable tool in the evaluation of exercise capacity and ventilatory efficiency in patients with COPD and HF.^15^ Several CPET indices and ventilatory parameters are known to be good metrics for the magnitude of impairment in CRF and prognosis of patients with various conditions including COPD and HF.^9^ Peak VO_2_ is a well-known independent predictor of disease severity and prognosis for cardiac-related events in patients with HF, a high-risk population.^15,16^ In addition, peak VO_2_ has been shown to be a better predictor of mortality and quality of life compared with FEV_1_ in patients with COPD.^17^ The presence of concomitant COPD and HF was associated with significantly lower peak VO_2_ in the current study, with the COPD–HF group achieving the lowest peak VO_2_ of 19.93 (95% CI: 18.60, 21.27) mL/kg/min. Maximal workload has been found to be a powerful predictor of 1-year mortality and urgent cardiac transplantation in a population of 226 patients with HF.^18^ Similarly, lower maximal workload in a population of 609 patients with moderate to severe emphysema was found to be a strong independent predictor of survival.^19^ This real-life study supports these previous publications in a large cohort of unselected patients referred for CPET.

Beyond the severity of impairment in CRF in this population, we observed numerous differences between parameters of ventilatory efficiency mediated by the coexistence of COPD and HF. The VE/VCO_2_ slope has been a useful prognostic marker in patients with HF and in most studies a stronger predictor of mortality than peak VO_2_.^16,20–23^ A VE/VCO_2_ slope higher than 32.8 units in patients with HF has been associated with an increase in mortality in a meta-analysis by Poggio *et al*.^24^ Similarly, in a retrospective analysis performed in patients with COPD prior to lung resection for lung cancer, an increased VE/VCO_2_ slope was a significant independent predictor of mortality post-operatively.^25^ In FRIEND, patients with COPD or HF had a statistically significant higher VE/VCO_2_ slope than controls, with the highest slope observed in patients with both conditions [36.73 (95% CI: 35.78, 37.68) units]. This marked increase represents a significant ventilatory inefficiency in these high-risk patients.

Abnormal resting PETCO_2_ and peak exercise PETCO_2_, which reflect the integrity of cardiac function as well as lung ventilation and perfusion, are both associated with a deterioration of functional, ventilatory, and cardiac performances in response to exercise.^23,26^ In a study by Paoletti *et al.*^27^, higher PETCO_2_ values were associated with advanced underlying emphysema and airway obstruction in patients with COPD and thus may be a marker of disease severity. In another clinical investigation, patients with COPD exhibited a steady increase in PETCO_2_ mediated to a rise in airway resistances.^28^ The lower PETCO_2_ values in patients with COPD in our cohort could be attributed to less severe disease as evidenced by higher FEV_1_/FVC ratios (0.638 ± 0.002 vs. 0.42 ± 0.08) in our study compared with the study by Paoletti *et al.*^27^ Lower alveolar PCO_2_ from alveolar hyperventilation combined with ventilation of poorly perfused alveoli likely explains the lower PETCO_2_ values observed in the clinical syndrome of COPD–HF.^29^

Our overall results are in agreement with the observations from Guazzi *et al.*^30^ who reported CPET characteristics in 69 subjects with COPD and HF matched to 69 patients with HF alone. Similar to our findings, this small investigation described a significant decrease in CRF in subjects with coexisting COPD and HF and significant abnormalities in all CPET parameters. Our results were derived from a much larger number of patients and yielded similar conclusions, although our population had higher CRF based on peak VO_2_ reported. In another small prospective study Arbex *et al.*,^31^ 98 patients with moderate to severe COPD with coexisting HF (LVEF < 50%) underwent CPET. Compared with COPD controls, patients with both HF and COPD had lower peak exercise capacity, greater ventilatory inefficiency, and lower PETCO_2_. They also reported that the major limiting symptom to reach maximal exercise in the patients with COPD with HF was leg fatigue compared with dyspnoea in the COPD only group. Thus, we may speculate that patients with COPD and HF may exhibit some impairment in oxygen delivery to the exercising muscle, increased muscle hypoxia, and consequently greater muscular deconditioning.

The results of this study should be interpreted in the context of its significant limitations. Although FRIEND the largest data set investigating COPD and HF, the study was retrospective and observational in nature, making it impossible to rule out the influence of any unmeasured confounding variables. The nature of the database also prevents us from accessing detailed clinical information on patients that may have been helpful to better understand and characterize the CRF impairment associated with different CPET parameters. Potentially important yet not computed parameters include HF phenotypes, the severity of LV dysfunction and/or COPD, the presence and severity of pulmonary hypertension, and detailed information on medications such as beta-adrenergic blockers and bronchodilators. Another limitation specific to the FRIEND database is the small yet significant age difference between the four groups. While this could account for the observed differences in CRF in the COPD–HF group, the variation in peak VO_2_ is well above the expected decline of ∼10% per decade that can be attributed to normal ageing, regardless of patients’ activity level.^32^ Finally, outcomes were not tracked in FRIEND. As such, the clinical consequences of these findings could not be assessed and require further investigation.

## Conclusions

Patients referred for CPET with COPD and concomitant HF exhibit a profound impairment in CRF compared with patients with COPD or HF alone. Early identification of pulmonary obstruction in patients with HF by more frequent usage of pulmonary function testing may contribute to providing better treatment for both COPD and HF in these high-risk individuals.

## Data Availability

The data underlying this article will be shared on reasonable request to the corresponding author.

## Lead author biography

Dr Jacinthe Boulet MDCM, MPH, completed her medical school and internal medicine residency at McGill University. She completed her cardiology residency at the Université de Montréal and her Master of Public Health at the Harvard T.H. Chan School of Public Health as a research fellow affiliated with the Brigham and Women’s Hospital and Harvard Medical School. She completed her clinical fellowship in advanced HF and heart transplantation at the Brigham and Women’s Hospital and is now a faculty member of the Montreal Heart Institute in the capacity of a clinician-scientist as an advanced HF and heart transplantation specialist.
